# Aurora B kinase inhibitor AZD1152: determinants of action and ability to enhance chemotherapeutics effectiveness in pancreatic and colon cancer

**DOI:** 10.1038/bjc.2011.21

**Published:** 2011-02-08

**Authors:** A Azzariti, G Bocci, L Porcelli, A Fioravanti, P Sini, G M Simone, A E Quatrale, P Chiarappa, A Mangia, S Sebastian, D Del Bufalo, M Del Tacca, A Paradiso

**Affiliations:** 1 Clinical Experimental Oncology Laboratory, National Cancer Institute, Via Hahnemann 10, Bari 70126, Italy; 2 Department of Internal Medicine, Division of Pharmacology and Chemotherapy, University of Pisa-Scuola Medica, Via Roma, 55, Pisa 56126, Italy; 3 Cancer and Infection Research Area, AstraZeneca, Alderley Park, Macclesfield, UK; 4 Experimental Chemotherapy Laboratory, Regina Elena Cancer Institute, Via delle Messi d’Oro 156, Rome 00158, Italy; 5 Clinical Pharmacology Centre for Drug Experimentation, Azienda Ospedaliera Universitaria Pisana, Via Roma 67, Pisa 56126, Italy

**Keywords:** AZD1152, gemcitabine, oxaliplatin, colon cancer, pancreatic cancer

## Abstract

**Background:**

AZD1152, the prodrug for AZD1152-hydroxyquinazoline pyrazol anilide (HQPA), is a selective inhibitor of Aurora B kinase activity. Preclinical evaluation of AZD1152 has been reported in several human cancer models. The potentiality of this compound in combination therapy warrants further investigation in solid tumours.

**Experimental design:**

This study explored the effects of AZD1152-HQPA in colon and pancreatic tumour cells. The antitumour properties of AZD1152, either as single agent or in combination with chemotherapeutics, were evaluated in each study model. The efficacy and the toxicity of AZD1152 alone and in combination with gemcitabine were validated in pancreatic tumour xenograft model.

**Results:**

AZD1152-HQPA treatment resulted in a dramatic increase of chromosome number, modification of cell cycle and induction of apoptosis. The most effective combination was that with chemotherapeutics given soon after AZD1152 in both tumour cell types. The effectiveness of the sequential schedule of AZD1152 with gemcitabine was confirmed in nude mice bearing MiaPaCa-2 tumours, showing inhibition of tumour volumes and delaying of tumour growth after the interruption of the treatments.

**Conclusion:**

Here we show that AZD1152-HQPA enhances oxaliplatin and gemcitabine effectiveness in colon and pancreatic cancer, respectively. First, we provide advances into administration schedules and dosing regimens for the combination treatment in *in vivo* pancreatic tumour.

The Aurora family comprises three related kinases, namely A, B and C; expression of the first two is closely linked to the proliferation of many cell types, although the role of the third one is not yet clearly defined (Katayama *et al*, 2003). Increased cellular levels of these kinases may be related to genetic instability and is evident in various cancer types including breast, ovarian, colon and pancreatic. Aurora A kinase is located in the pericentriolar region where its function is related to centrosome function and duplication, mitotic entry and bipolar spindle assembly (Lukasiewicz and Lingle, 2009). During its activity in metaphase, the kinase migrates to mitotic spindle poles and mid-zone microtubules, phosphorylating and recruiting several microtubule-associated proteins to the centrosome to promote maturation. Aurora B, together with survivin, INCENP and borealin, forms the chromosomal passenger complex and acts as its catalytic component allowing (i) correct segregation of chromatids at mitosis, (ii) histone H3 phosphorylation, an event probably related to chromosome condensation and (iii) cytokinesis (Vogt *et al*, 2009). Aurora C kinase appears to have a role similar to that of Aurora B kinase, but its expression appears to be restricted to the testis (Sen, 2009).

All these characteristics make this family of kinases an attractive target for tailored anticancer therapy.

Several small-molecule inhibitors of Aurora kinases have been developed and are currently in early clinical evaluation, including AZD1152 (Schellens *et al*, 2006; Boss *et al*, 2009). AZD1152 is a dihydrogen phosphate prodrug of a pyrazoloquinazoline Aurora kinase inhibitor (AZD1152-hydroxyquinazoline pyrazol anilide (HQPA)) that is rapidly converted into the active moiety AZD1152-HQPA following parental administration *in vivo*. AZD1152-HQPA is a highly potent and selective inhibitor of Aurora B (*K*_i_ 0.36 nmol l^−1^) compared with Aurora A (*K*_i_ 1.3669 nmol l^−1^) and has high selectivity *vs* a panel of 50 other kinases (Keen *et al*, 2005).

In several preclinical models, AZD1152 has shown significant inhibition of tumour growth both as a monotherapy and in combination with ionising radiation and chemotherapeutic agents including vincristine, daunorubicin and CPT-11 (Wilkinson *et al*, 2007; Yang *et al*, 2007; Evans *et al*, 2008; Tao *et al*, 2008; Walsby *et al*, 2008; Ikezoe *et al*, 2009; Nair *et al*, 2009a, 2009b; Oke *et al*, 2009).

In 2006 ASCO Annual meeting and in 2010, Schellens and co-workers presented preliminary results of a phase I clinical trial performed with AZD1152 in patients with advanced solid tumours (Schellens *et al*, 2006; Boss *et al*, 2010). They reported significant disease stabilisation suggesting a future promising clinical development for this agent, which showed maximum-tolerated doses (MTDs) of 200 and 450 mg, DLT of 450 mg, significant non-haematological toxicities and a clearance of 22.4±1.03 l h^−1^. Another clinical trial, a phase I/II, open-label two-part study, provided information on the safety and efficacy of AZD1152 in patients with advanced acute myeloid leukaemia showing that this agent had an acceptable tolerability profile in patients with acute myeloid leukaemia (AML) (Lowenberg *et al*, 2009). The MTD of AZD1152, given as a continuous 7-day infusion every 21 days, was 1200 mg and the overall clinical response rate was of 23%. Currently, AZD1152 is being evaluated in phase II trials as monotherapy or in combination with low-dose cytosine arabinoside for the treatment of elderly patients with AML who are unsuitable for standard induction treatment (www.clinicaltrials.gov).

The main open questions in considering Aurora kinase inhibitors as promising anticancer drugs are the possible predictive factors for response and their combination with other cytotoxic agents.

These topics are explored only limitedly, and among possible predictive factors, it was suggested CHFR, a mitotic checkpoint protein and p53, the inactivation of which leads to increased sensitivity to this class of drugs (Nair *et al*, 2005; Summers *et al*, 2005; Yu *et al*, 2005). For what concerns data on Aurora kinase inhibitors in combination with chemotherapeutic drugs, more literature evidences are available, in particular with daunorubicin, SN-38, vinorelbine, gemcitabine, docetaxel, oxaliplatin and 5-fluorouracil (Nair *et al*, 2004; Soncini *et al*, 2006; Hoover, 2007).

However, the exploration of mechanisms responsible for Aurora kinase inhibition, its regulation, downstream effectors and upstream regulators after AZD1152 administration provides a rapid progress in understanding the complex mechanism of action of this relatively new class of drugs that enlighten their utilisation in clinical practice.

Our interest lies in the use of Aurora kinase inhibitors in combination with chemotherapy in solid tumours and experiments reported in this paper were carried out in colon and pancreatic cancer cells.

The selection of *in vitro* models is justified by the high levels of Aurora kinases, related to genetic instability (Kimura *et al*, 1999), in a wide range of human cancers, including colorectal and pancreatic cancers (Katayama *et al*, 1999; Warner *et al*, 2006).

Advances in chemotherapy have led to changes in treatment practice standards and improved survival for colorectal cancer patients. Platinum-based chemotherapeutics, camptothecins and 5-FU, remain the cornerstone chemotherapy drugs for colorectal cancer; however, today they are administered as part of multidrug regimens (Muggia and Fojo, 2004; Hegde *et al*, 2008; Cheung *et al*, 2009). New targeted therapies offer great promise against colorectal cancer. These therapies, including drugs and monoclonal antibodies, target specific biological processes used by cancers to grow and spread. Recent evidence has indicated that the induction of apoptosis in human colon carcinoma cell system take advantage from response to various antimitotic drug treatments, enforcing the idea that targeting the mitotic kinases, such as Aurora B, is a viable area of research for the exploitation of novel anticancer strategies in the treatment of colorectal cancer patients.

In the literature, Nair and co-workers have already reported the possibility to combine AD1152 with campothecins in *in vitro* and *in vivo* colorectal cancer model (Nair *et al*, 2009a), and we decided to confirm this hypothesis by investigating the effectiveness of this drug in combination with the other conventional drug, the oxaliplatin.

For what concerns pancreatic tumour, it is well known that it is difficult to treat and is associated with a dismal prognosis. This tumour spreads rapidly and, because it is difficult to detect in the early stages of disease, most patients are not diagnosed until they have metastatic disease. Consequently, the 5-year survival rate is less than 2% (Wong and Lemoine, 2009) and there is a need for novel therapeutic approaches based on the targets involved in pancreatic carcinogenesis, including both signal-transduction and embryonic development pathways. Recent pre-clinical studies on novel therapeutic approaches have provided promising results through multiple inhibition of the same pathway or through the inhibition of multiple signalling pathways. These pharmacological approaches should prevent the development of escape or resistance mechanisms. The standard chemotherapeutic drug for patients with advanced pancreatic cancer is gemcitabine, an antimetabolite that is incorporated in the DNA to interfere with the process of division of cancer cells, thus hindering their growth. The limited encouraging data from clinical evaluation of gemcitabine in association with chemotherapy or target-oriented drugs, mainly inhibitors of tyrosine kinase receptor pathways, suggest that a promising therapeutic approach could be enhanced inhibition of mechanisms responsible for DNA synthesis, repair and for correct cytokinesis by combining gemcitabine with Aurora kinase inhibitors (Moufarij *et al*, 2003; Bayraktar *et al*, 2010).

Thus, we evaluated whether AZD1152 could enhance the efficacy of gemcitabine in a pancreatic cancer *in vitro* model, suggesting the molecular pathways that are activated and required for treatments efficacy. Moreover, we decided to *in vivo* validate the promising antiproliferative results of the combination in pancreatic tumour xenografts; in our opinion, to gain further knowledge on the possibility to use Aurora kinase inhibitors in solid tumours, treatment could send toward a new approach for cancer therapy, and since in literature the validation of *in vitro* results of this drug in multitherapy in colorectal cancer was already available (Nair *et al*, 2009a), we focused on the other cancer model.

## MATERIALS AND METHODS

### Drugs and chemicals

AZD1152-HQPA and AZD1152 were provided by AstraZeneca Pharmaceuticals (Macclesfield, UK). Stock solutions of AZD1152-HQPA were prepared at 20 mM in DMSO and stored in aliquots at −20°C. Gemcitabine (Gemzar) and oxaliplatin (Eloxatin) were provided by Eli Lilly and Co. (Indianapolis, IN, USA) and Sanofi-Synthelabo (Milan, Italy) respectively. Further dilutions were made in the medium supplemented with 10% foetal bovine serum, 2 mM glutamine, 50 000 U l^−1^ penicillin and 80 *μ*M streptomycin. For *in vivo* studies, sterile AZD1152 powder was dissolved in Tris buffer 0.3 M, pH 9.0, to obtain a solution at the concentration of 25 mg ml^−1^. Gemcitabine (Eli Lilly and Co.) was diluted in sterile saline solution for *in vivo* use.

### Cell lines

The colon cancer (HCT116 and Colo205) and pancreatic (MiaPaCa-2) cell lines were kindly provided by Professor M Coluccia (University of Bari, Bari, Italy) and obtained from the American Type Culture Collection (ATCC, Manassas, VA, USA), respectively. Cells were cultured *in vitro* in RPMI supplemented with 10% foetal bovine serum, 2 mM glutamine, 50 000 U l^−1^ penicillin and 80 *μ*M streptomycin in a humidified incubator at 37°C with an atmosphere containing 5% CO_2_.

### Cell imaging

Cells incubated with 30 and 300 nM AZD1152-HQPA for 1–3 days were analysed by light inverted microscopy.

### Cell proliferation assay

Determination of cell growth inhibition was performed using the 3-[4,5-dimethylthiazol-2-yl]-2,5-diphenyltetrazoliumbromide (MTT) assay and by cell counting. The MTT assay for each concentration responsible for 50% inhibition of cell growth (IC_50_) determination and drug combination effectiveness was performed as described in (Azzariti *et al*, 2004).

For cell count determination, 1.5 × 10^5^ cells were plated in 35 mm Petri dishes, exposed to drug(s), harvested in trypsin and counted. For IC_50_ determination, AZD1152-HQPA was given at concentrations of 3, 30, 300 nM, 3 and 30 *μ*M for 3 days. The IC_50_ was defined as the drug concentration yielding a fraction of affected (no surviving) cells=0.5, compared with untreated controls and was calculated utilising the CalcuSyn ver.1.1.4 software (Biosoft, Cambridge, UK). In the combination studies, AZD1152-HQPA was given at 30 and 300 nM and the chemotherapeutic agents at the concentration reported in each experiment. To define the best schedule for the combination, either simultaneous or sequential utilisation of the two drugs were tested. Each experiment was carried out in triplicate.

### Cell cycle analysis

Cells were harvested, washed twice in ice-cold PBS (pH 7.4), fixed in 4.5 ml of 70% ethanol at −20°C and washed once in ice-cold PBS. The pellet was resuspended in PBS containing 1 mg ml^−1^ RNase, 0.01% NP40 and the cellular DNA was stained with 50 *μ*g ml^−1^ propidium iodide (PI) (Sigma, St Louis, MO, USA). Cells were stored in ice for at least 1 h before analysis. Cell cycle determinations were performed using a FACScan flow cytometer (Becton Dickinson, Franklin Lakes, NJ, USA), and data were interpreted using the CellQuest software, provided by the manufacturer.

### Cell apoptosis assay

Apoptosis was determined by dual staining with Annexin V-FITC and PI using the Annexin V-FITC detection kit (BD Transduction) and by PI following the manufacturer's protocol and analysed by flow cytometry with FACScan (Becton Dickinson). Apoptosis was also determined using BAK-epitope, an early marker of apoptosis. HCT116 cells were seeded in 96-well plates and 24 h later subjected to different dosing schedules (concurrent or sequential). Cells were then stained for cell number by Hoechst (nuclei), and apoptosis by BAK. Images were taken and end points quantified using the Cellomics Arrayscan II platform (Cummings *et al*, 2008).

### Chromosome number determination

Cells were treated with colcemid 0.5 *μ*M for 4 h, harvested, washed twice in PBS and swelled in hypotonic solution (0.075 M potassium chloride (KCl)) for 10 min at room temperature. Cells were fixed with methanol and acetic acid (3:1), dropped on slides and left to dry for 24 h. Chromosomes were stained with quinacrine 5% and analysed using a fluorescence microscope (Olympus BX40). A number of at least 50 metaphases for each specimen were evaluated.

### Western blot analysis

Protein extracts were obtained by homogenisation in RIPA buffer (0.5 M NaCl, 1% Triton X-100, 0.5% NP40, 1% deoxycolic acid, 3.5 mM SDS, 8.3 mM Tris–HCl, pH 7.4, 1.6 mM Tris base) and treated with 1 mM phenylmethylsulphonyl fluoride. Total proteins were measured and analysed as described in Azzariti *et al* (2004).

In particular, 50 *μ*g were electrophoretically separated on 10% acrylamide gel (SDS–PAGE by Laemmli). Signal was detected by chemoluminescence assay (ECL-Plus, Amersham Life Science, Little Chalfont, UK). Expression levels were evaluated by densitometric analysis using the Quantity One software (Biorad, Hercules, CA, USA) and *β*-actin expression levels were used to normalise the sample values.

### Antibodies

The monoclonal antibody anti-Akt (no. 9272), anti-phospho-Akt (Ser473) (no. 9271), anti-p53(DO-1) (Sc-126) and anti-*β*-actin AC-15 (A5441) were provided by Cell Signalling (Milwaukee, WI, USA), Santa Cruz Biotechnology (Santa Cruz, CA, USA) and Sigma-Aldrich, respectively. A mouse-HRP and a rabbit-HRP (Amersham Pharmacia Biotech, Uppsala, Sweden) were used as secondary antibody. All antibodies were utilised at the recommended dilutions.

### Fluorescence immunocytochemistry

Cells were seeded onto coverslips. After overnight incubation, they were fixed in 3.7% paraformaldehyde, washed and permeabilised with 0.1% Triton X-100. After saturation with 0.1% gelatin in PBS, cells were subsequently immunostained overnight with phospho-specific histone H3 (Ser10) antibody (Upstate, New York, NY, USA). Cells were then incubated with FITC-conjugated secondary antibody (Becton Dickinson) for 1 h. Nuclei were counterstained with 0.5 lg ml^−1^ 4′,6-diamidino-2-phenylindole (DAPI). The images were captured using a fluorescence microscope (Olympus BX40), equipped with × 20 objective with a SenSys 1401E-Photometrics charge-coupled device camera. FITC was excited using the 488 laser line and DAPI using the 568 laser line.

### *In vivo* experiments

*Animals.* CD *nu*/*nu* male mice weighing 20 g were supplied by Charles River (Milan, Italy) and were allowed unrestricted access to food and tap water. Housing and all procedures involving animals were performed according to the protocol approved by the Academic Committee for the animal experimentation of the University of Pisa, in accordance with the European Community Council Directive 86-609, recognised by the Italian government, on animal welfare.

*MiaPaCa-2 xenografts in *nu*/*nu* mice and drug treatments.* MiaPaCa-2 cell viability was assessed by Trypan blue dye exclusion. On day 0, 1.3 × 10^6^±5% cells per mouse were inoculated subcutaneously between the scapulae in 0.2 ml per mouse of culture medium without FBS. Animal weights were monitored and, upon appearance of a subcutaneous mass, tumour dimensions were measured every 4 days in two perpendicular directions using callipers. Tumour volume (mm^3^) was defined as follows: ((*w*1 × *w*2 × *w*2) × (*π*/6)), where *w*1 and *w*2 were the largest and the smallest tumour diameter (mm), respectively. The mice were randomised into four groups of eight animals.

To treat an established tumour (∼100 mm^3^), from day 15 after cell inoculation AZD1152, gemcitabine and their respective vehicles were administered intraperitoneally to mice as follows: group (1) AZD1152 25 mg kg^−1^ for 4 consecutive days, followed by sterile saline (gemcitabine vehicle); group (2) solution of Tris buffer 0.3 M alone (AZD1152 vehicle) for the first 4 days, followed, on day 5, by gemcitabine 120 mg kg^−1^ four times a day at 3-day intervals (Bocci *et al*, 2005); group (3) combination treatment of AZD1152 25 mg kg^−1^ for 4 consecutive days, followed by gemcitabine 120 mg kg^−1^ four times a day at 3-day intervals; and group (4) AZD1152 vehicle alone, followed by gemcitabine vehicle alone (control group). After treatment, the mice were observed for 10 days and then killed by an anaesthetic overdose. Moreover, the %*T*/*C* value has been calculated as follows (Bocci *et al*, 2008): (mean tumour volume of the treated group/mean tumour volume of the control group) × 100.

### Statistical analysis

All *in vitro* experiments were performed in triplicate, and results have been expressed as the mean±standard deviation (s.d.), unless otherwise indicated. Statistical differences of *in vitro* and *in vivo* data were assessed by ANOVA, followed by the Student–Newman–Keuls test. *P*-values lower than 0.05 were considered significant. Statistical analyses were performed using the GraphPad Prism software package version 5.0 (GraphPad Software Inc., San Diego, CA, USA).

## RESULTS

### AZD1152-HQPA inhibits cell growth in the function of drug concentration and exposure time

**Figure 1:**
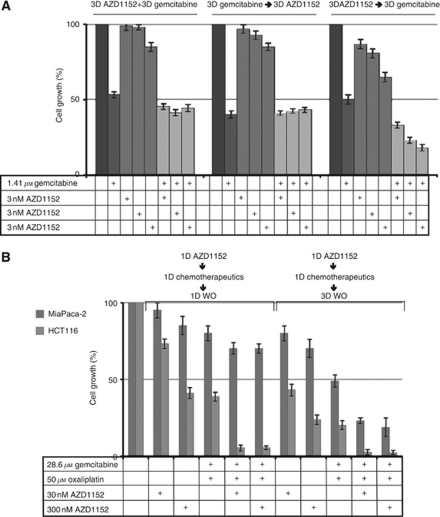
Drug(s)-dependent cell growth inhibition. Cells were incubated with AZD1152-HQPA (AZD) and/or oxaliplatin (oxa) or gemcitabine (gem) with continuous or intermittent exposure. Cell survival was determined by cell counts. In (**A**) and (**B**), results from MiaPaCa-2, and MiaPaCa-2 and HCT116. 1D WO=1 day drug washout; 3D WO=3 day drug washout.

In initial experiments, AZD1152-HQPA, at concentrations ranging between 3 nM and 3 *μ*M, given for 1 day, followed by 0–5 days wash out, failed to show a strong reduction of cell growth by MTT assay despite the clear effects obtained by cell counts shown in Figure 1. The experimental conditions have been reported in a previous analysis (Cummings *et al*, 2008) and our results suggested that the MTT assay underestimated the effects of AZD1152-HQPA on cell number, possibly because the cells increase in size before undergoing apoptosis. Thus, the following experiments were designed to evaluate the effectiveness of AZD1152-HQPA alone and in combination with conventional chemotherapeutic agents, by direct cell counting.

Of the three cancer cell lines analysed (HCT116, Colo205 and MiaPaCa-2), the HCT116 cell line was the most sensitive to AZD1152-HQPA treatment. The effectiveness of AZD1152-HQPA increased as a function of concentration and time exposure in all cell lines. In detail, 1-day incubation of HCT116 cells with AZD1152-HQPA 30 nM induced a reduction in cell numbers of approximately 20, 30 and 80% after 0, 2 and 4 days drug wash out, respectively (Figure 1B). In the same experimental conditions, treatment of Colo205 cells reduced cell numbers by approximately 15, 20 and 40%, respectively (data not shown). The effects on cell growth were greater at higher drug concentrations or after longer exposure to the drug. For example, treatment with AZD1152-HQPA 300 nM increased cell growth inhibition after 2 days wash out from 40 to 85% and from 40 to 55% in HCT116 and Colo205 cells, respectively. Conversely, treatment with AZD1152-HQPA for just 1 day induced only a slight inhibition in MiaPaCa-2 cell growth, which increased with washout time exposure from 5 to 40% at 0 and 5 days after wash out, respectively.

The choice of chemotherapeutics for the combination experiments was suggested by both their conventional utilisation in cancer chemotherapy and mechanisms of action, interacting with our Aurora B kinase inhibitor, that is, oxaliplatin and gemcitabine in colon and pancreatic cancer *in vitro* models, respectively. To define the best schedule for drug combination, AZD1152-HQPA was administered before, together or after the chemotherapeutic drugs. Furthermore, pilot kinetic experiments were performed to find the most effective combination with AZD1152-HQPA after 1 day and 3 days. The conventional chemotherapeutics were utilised at IC_50_ concentrations; oxaliplatin was given for 1 day at 50 and 35 *μ*M in HCT116 and Colo205 cells, respectively; gemcitabine was given for 1 day at 28.6 *μ*M, followed by 3 days wash out or for 3 days at 1.41 *μ*M in MiaPaCa-2 cells. IC_50_ concentrations were lower than plasma concentrations in treated patients for gemcitabine. Conversely, the oxaliplatin concentration was higher than plasma concentrations, but compatible with its concentration in animal model for 1-day oxaliplatin administration (Ostberg *et al*, 2008; Veltkamp *et al*, 2008). Initially, we determined the best schedule of combined administration of AZD1152-HQPA plus gemcitabine or oxaliplatin in both *in vitro* models and in Figure 1A we reported data from MiaPaca-2 cells, which are representative of those obtained in colon cancer cells. The comparison of the three different schedules indicates that the best schedule is one in which the AZD1152 is given before the chemotherapeutic agent and is independent of AZD1152-HQPA time exposure. Moreover, AZD1152-HQPA appeared to enhance the effectiveness of gemcitabine in a concentration-independent manner between 3 and 300 nM in these experimental conditions (Figure 1A). The evaluation of the ability of AZD1152-HQPA to enhance chemotherapeutics cytotoxicity was also measured after giving the two drugs in the most promising schedule, followed by 1–3 days wash out, and, as evident in Figure 1B, cell growth inhibition increased as a function of time. In Figure 1B, all results of AZD1152-HQPA in combination with oxaliplatin or gemcitabine after 3 days wash out were statistically different from each chemotherapeutic alone (*P*-value <0.05); conversely, at shorter time wash out, only in HCT116 cells, data were statistically different.

### AZD1152-HQPA perturbs cell cycle progression

**Figure 2:**
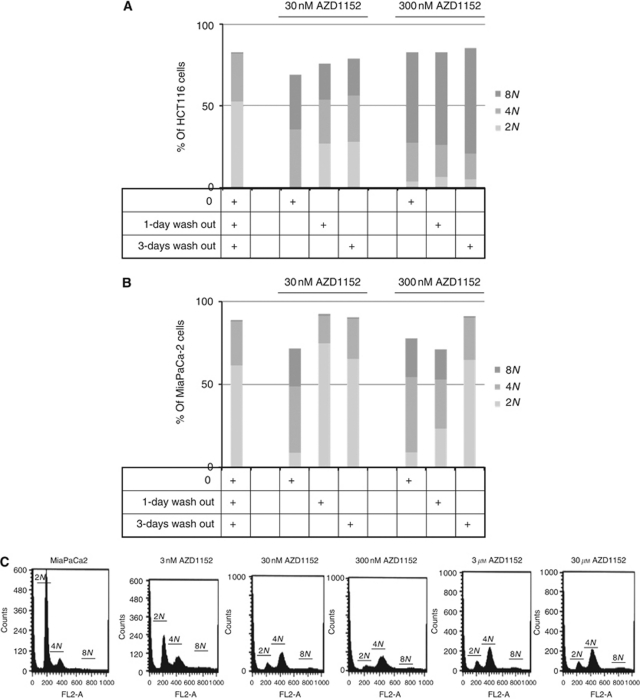
AZD1152-HQPA-dependent cell cycle modification. Cells were incubated with AZD1152-HQPA (AZD) for 1 day, followed by 0–3 days drug wash out, and then the cell cycle was analysed by CFM as described in Material and Methods section. In (**A**) and (**B**), results from AZD-treated HCT116 and MiaPaCa-2 cells, followed by 0, 1 and 3 days drug wash out were reported. In (**C**), results from 3-day AZD-treated MiaPaCa-2 cells are shown in the function of concentration. FL2-A=FL2-area (total cell fluorescence).

A strong perturbation of cell cycle progression may be responsible for the observed reduction in cell growth and this hypothesis has been investigated in our model. In HCT116 cells, 1-day exposure to 30–300 nM AZD1152-HQPA induced an additional round of DNA synthesis such that cells became >8*N* and the effect was partially maintained during a 1–2 days drug washout period, as reported in Figure 2A. The behaviour was similar in both colon cancer cell lines (data not shown). In contrast, in MiaPaCa-2 cells, AZD1152-HQPA at both 30 and 300 nM induced cell cycle arrest in mitosis (4*N*) soon after 1-day treatment, which was removed following drug wash out (Figure 2B). To investigate whether the recovery of cell cycle progression was due to intrinsic resistance of cells, induction of apoptosis or simply a reduction of AZD1152-HQPA concentration during the washout period, experiments were performed by exposing MiaPaCa2 cells to AZD1152-HQPA for 3 days. These experiments showed that the effects of the drug on cell cycle distribution remained stable over a longer treatment period. These findings suggest that reversal of the effects of AZD1152-HQPA after shorter treatment times is due to reduction in drug concentration rather than a resistance mechanism or induction of apoptosis because of the absence of a sub-G0 peak (Figure 2C). The behaviour was similar in all cancer cell lines utilised (data not shown). In conclusion, AZD1152-HQPA induced a marked concentration-dependent G2/M-phase cell (4*N*) accumulation associated with increased DNA content in all cell lines, which was dependent on drug concentration. This phenomenon was reversed with increasing time of drug wash out. Furthermore, cell cycle analysis was carried out in the combination study in the colon cancer model in which AZD1152-HQPA was given with oxaliplatin, which is known to block cells in G2/M phase. Results showed a higher accumulation of cells with 4*N* DNA content with a concomitant increase of cell death (data not shown).

### AZD1152-HQPA strongly modifies cellular morphology and induces endoreduplication

**Figure 3:**
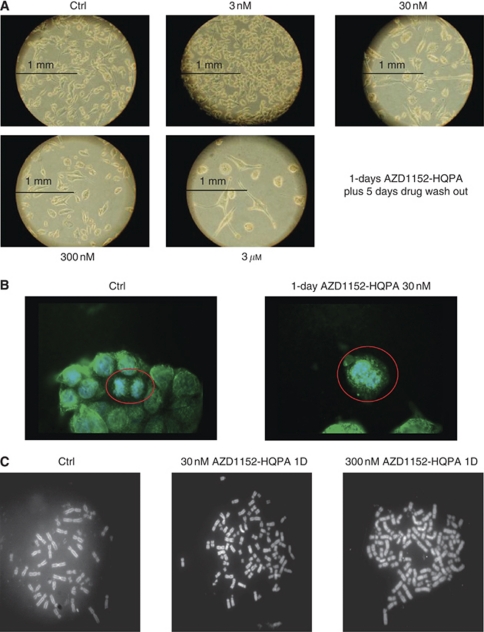
Morphological changes by AZD1152-HQPA exposure. (**A**) Increase in MiaPaCa-2 cell size induced by AZD1152-HQPA exposure at various concentrations and wash out. (**B**) Chromosome alignment and segregation prevented by AZD1152-HQPA in MiaPaCa-2 cells induced abnormal prometaphase detected by immunofluorescence (tubulin staining in green and DAPI in blue). (**C**) MiaPaCa-2 cells were incubated with AZD1152-HQPA for 1 day and the chromosome number was determined by quinacrine assay utilising fluorescence microscopy, as described in Materials and Methods section. A number of at least 50 metaphases for each specimen were evaluated.

All experiments evidenced major modification in cell structure and in cell growth features after cell exposure to AZD1152-HQPA. The effect of treatment with 30 and 300 nM AZD1152-HQPA for 1–5 days on cell morphology was determined by light microscopy and showed major modifications in cell structure and in cell growth features, in all cell lines. In particular, treated cells were larger than untreated cells and this was confirmed as an increase in forward light scatter during flow cytometric cell cycle analysis; in Figure 3A results from MiaPaCa-2 cells are shown. The effects of AZD1152-HQPA on chromosomal localisation and structure were examined by immunofluorescence. These experiments showed expected abnormalities in chromosomal localisation at the centromere and mitotic spindles, together with endoreduplication associated with inhibition of cytokinesis. Data from the MiaPaCa-2 pancreatic cell line are reported in Figure 3 and showed a strong induction of endoreduplication, as a function of drug concentration and time exposure, and abnormal prometaphases (Figure 3B). To further confirm the ability of AZD1152-HQPA to enhance polyploidia and to inhibit chromosome alignment and segregation, chromosome number and localisation were evaluated. Cells were treated with 30 and 300 nM AZD1152-HQPA for 1 day with or without a subsequent 1–3 days wash out. Treated cells displayed a strong increase of chromosome number of approximately two- to four-fold greater than in control (untreated) cells after only 1-days treatment (Figure 3C), which was particularly evident at higher concentrations (300 nM AZD1152-HQPA) in both colon and pancreatic cancer cell lines, also shown by a substantial increase of >4*N* subpopulations on flow cytometry analysis.

### Concurrent treatment of AZD1152-HQPA with oxaliplatin enhances apoptosis in colon cell line

**Figure 4:**
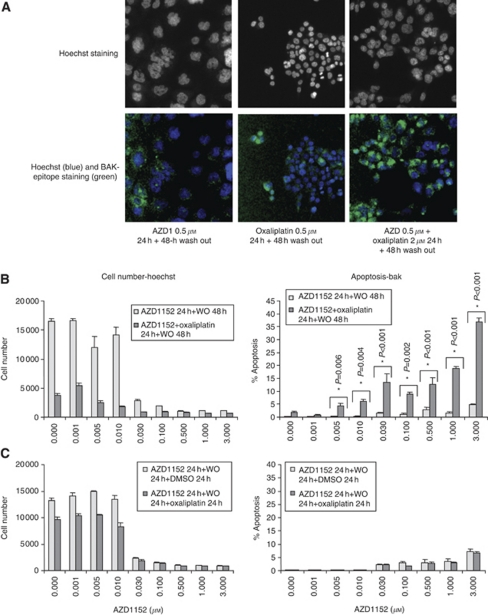
Effect of AZD1152-HQPA in combination with oxaliplatin on cell growth and apoptosis. HCT116 cells were seeded in 96-well plates. After 24 h, cells were subjected to concurrent dosing schedule (**A** and **B**) or a sequential dosing schedule (**C**). Cells were then stained for cell number by Hoechst and BAK-epitope, an early marker of apoptosis. Images were taken and end points quantified using the Cellomics Arrayscan II platform. Graphs are representative of three independent experiments. Columns, mean; bars, s.e. ^*^P<0.05 *vs* oxaliplatin 0 *μ*M (two-way ANOVA); WO=washout.

It has previously been shown that inhibition of Aurora B activity by treatment with AZD1152 induces failure of cell division, leading ultimately to apoptosis (Wilkinson *et al*, 2007). Here, we investigated the effect of AZD1152 in combination with oxaliplatin on cell survival and cell growth. *In vitro* treatment of AZD1152-HQPA, followed by drug wash out induced a significant cell growth arrest, but a minimal effect of apoptosis as measured by Annexin V or BAK staining (<10% of positive cells) (Figure 4). When cells were concurrently treated with AZD1152-HQPA and oxaliplatin, an enhanced inhibitory effect on cell growth and cell survival was observed compared with monotherapy (Figure 4). Two-way ANOVA statistical analysis of the data shows a significant difference in the mean values among the different concentrations of oxaliplatin (*P*<0.001) when simultaneously administrated with AZD1152-HQPA in HCT116 cells (Figure 4B). In comparison to monotherapy, where the two drugs are combined sequentially with 24 h drug wash out, an equal or small beneficial effect was observed (Figures 4B and C). These data suggest that the optimal schedule for combining AZD1152 and oxaliplatin within the time frame chosen for the experiments is concurrent treatment. An increase of 20% in apoptosis was observed when oxaliplatin was administered after AZD1152-HQPA, by Annexin V assay (data not shown). Importantly, none of the combination dosing schedules of AZD1152-HQPA with oxaliplatin resulted in an antagonistic effect compared with either monotherapy (data not shown).

### AZD1152-HQPA inhibits survival pathways as well as Aurora B kinase activity

**Figure 5:**
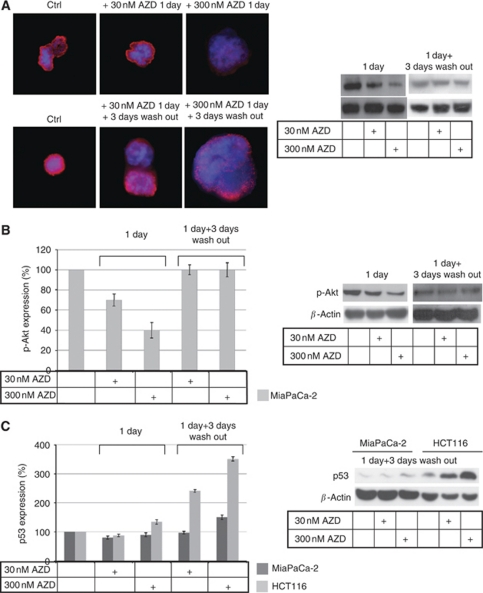
AZD1152-HQPA-dependent modulation of cell pathways. (**A**) MiaPaCa-2 cells were incubated with AZD1152-HQPA for 1 day, followed by 0 or 3 days drug wash out, and then histone 3 phosphorylation was analysed by immunofluorescence staining (Ser-10 pH3 staining in red and DAPI in blue) and western blotting. (**B** and **C**) Cells were incubated with AZD1152-HQPA and protein extracts were analysed by western blotting. The amount of p-AKT and p53 was determined using *β*-actin to normalise values.

The ability of AZD1152-HQPA to inhibit histone H3 phosphorylation, as a function of drug concentration, has been evaluated by immunofluorescence and western blotting analysis. Levels of phosphorylated histone H3 remained low during the drug washout time, showing that the Aurora B kinase activity was stably inhibited; in Figure 5A, results from MiaPaca-2 cells are reported and they are representative of all *in vitro* models. In addition to its specific cell target, survival and proliferation pathway modulation was also investigated, by determining Akt and Erk1/2 by western blotting. Results evidenced the absence of drug ability to modulate the total forms of both proteins (not shown). Conversely, decreased Akt phosphorylation was associated with drug treatment, probably because the cells are undergoing apoptosis; in MiaPaCa-2 cells, as reported in Figure 5B, p-Akt recovered the basic condition after 3 days drug wash out, a similar modulation was observed in colon cancer models, but the recovery, after 3 days wash out, reached at maximum 80% (data not shown). Furthermore, an increase in p53 was associated with AZD1152-HOPA treatment in colon cancer cell lines compared with the pancreatic cell line, in which its modulation is limited. These findings suggest that the increase in cell susceptibility is the start of the cell death process (Figure 5D).

### AZD1152, gemcitabine and their consecutive combination *in vivo*

**Figure 6:**
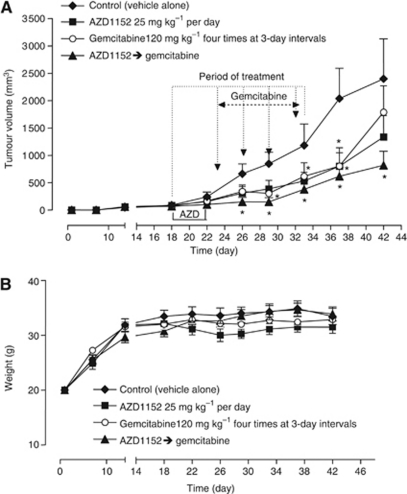
*In vivo* effects of AZD1152, gemcitabine and the sequential combination of AZD1152, followed by gemcitabine on MiaPaCa-2 tumour xenografts. (**A**) Antitumor effect of AZD1152, gemcitabine and their consecutive combination on MIAPaCa-2 tumours xenotransplanted in CD *nu/nu* mice. The mice were randomised into groups of eight animals. (**B**) Body weight of MIAPaCa-2 tumour-bearing control mice and mice treated with metronomic AZD1152, gemcitabine and their consecutive combination. Symbols and bars, mean±s.e.m.; ^*^P<0.05 *vs* vehicle-treated controls.

Our *in vitro* results suggest that AZD1152 could be an enhancer of the efficacy of gemcitabine in pancreatic tumour treatment and so we conducted *in vivo* studies to validate these findings. MiaPaCa-2 cells injected s.c. in CD *nu*/*nu* mice grew quite rapidly and tumour masses became detectable 7 days after xenotransplantation. Tumours in control animals showed a progressive enlargement in their dimensions and a marked growth after ∼30 days; a mean volume of ∼2500 mm^3^ was reached at day 42 when the animals of the control group were killed (Figure 6A). As expected, both gemcitabine and AZD1152 at the MTDs were able to significantly inhibit tumour growth in a similar extent as compared with controls, as well as their combination (Figure 6A). In the group of animals receiving the consecutive combination of AZD1152 and gemcitabine, the average reduction in tumour growth was more than double than the single drugs already by day 26 (e.g., the calculated *T*/*C* value was 22% of the combination *vs* 47% of AZD1152 alone and 51% of gemcitabine alone) and the tumour growth curve showed a marked delay after the interruption of the treatments if compared with the ones of single compounds, as shown in Figure 6A (at day 42, *T*/*C* value 34% of AZD1152+gemcitabine *vs* 56% of AZD alone and 75% of gemcitabine alone).

Figure 6B shows the toxicity profiles of the four different schedules. All the schedules were favourable and acceptable, with no loss of weight throughout the course of the treatment (Figure 6B). It is worth noting that the animals treated with the combination reached body weights similar to those treated with vehicles alone, confirming the safety of the association despite its higher therapeutic effect.

## DISCUSSION

There is considerable interest in combining novel targeted agents with standard chemotherapeutics to enhance efficacy. It is becoming increasingly obvious that the schedule of administration influences the success of these combination strategies (Azzariti *et al*, 2004, 2008; Bocci *et al*, 2005).

In the case of the emerging class of Aurora kinase inhibitors, studies on several promising drug combinations are currently in progress, such as taxane plus Aurora A RNA interference, MK-0457 plus etoposide or doxorubicin, docetaxel plus Aurora A short hairpin RNA and AZD1152 plus radiotherapy in various solid cancer models (Hata *et al*, 2005; Lee *et al*, 2006; Sun *et al*, 2007; Tanaka *et al*, 2007; Yang *et al*, 2007; Tao *et al*, 2008). Actually, several clinical trials are ongoing with this class of drug and briefly MK-0457, MLN8237, PHA-739358, PF-03814735 are in phase II (www.clinicaltrials.gov).

However, little is known about the mechanism of action of Aurora kinase inhibitors in combination with radio- and chemotherapy at the molecular level.

Our *in vitro* characterisation showed that AZD1152-HQPA monotherapy, the active form of the phosphate prodrug AZD1152, inhibited cell growth and induced apoptosis in colon and pancreatic cancer cell lines in a concentration- and time-dependent manner. The induction of apoptosis detected with the Annexin V and immunoflurescence assays was not reflected as an increase in a sub-G0/G1 peak population by flow cytometry. We believe that this is explained by the fact that DNA fragmentation associated with apoptosis occurs in a cell population that has undergone endoreduplication to become >4*N*. It has been suggested by Aihara *et al* (2009) that this makes detection of a sub-G0/G1 peak by conventional flow cytometry difficult. Data concerning the effects of AZD1152-HQPA on apoptosis are scant, although our analysis seems to be in disagreement with the results of Oke *et al* (2009), who reported that the Aurora B kinase inhibitor induced a strong increase in an Annexin V-positive cell fraction in studies in primary AML cells and cell lines. However, our experimental conditions are markedly different from those of Oke *et al* (2009) in that they involve prolonged drug exposure times, and it is likely that after a 1-day exposure, followed by 1-day drug washout period, the number of apoptotic cells that can be detected will be rather less than that seen after 4 days of continuous drug exposure. In addition, Oke *et al* (2009) suggest that most cells progress from polyploidy to apoptosis after prolonged drug exposure. In this, it appears that cells can maintain the ability to overcome polyploidy and can begin to divide after shorter exposure times. This finding suggests that 1-day exposure to AZD1152-hQPA might be sufficient to sensitise tumour cells to the effects of conventional cytotoxic agents, thereby reducing toxic effects. This ability to overcome cell cycle impairment and to recover the ability to enter mitosis seems evident in solid tumour cell lines, but not in an AML model, in which 48 h exposure to AZD1152-hQPA reduced the ability of cells to undergo clonal growth, when replated in fresh medium, suggesting a inverse correlation between recovery of cell growth ability and DNA increase (Walsby *et al*, 2008).

Our results are in agreement with those produced by Nair *et al* (2009a), who reported a small induction in apoptosis after 48 h treatment of HCT116 cells with AZD1152-HQPA. Our study is also in agreement with a recent study in which CPT-11 enhanced AZD1152 activity in a schedule-dependent manner (Nair *et al*, 2009a). In this study, the best schedule of administration was when the Aurora kinase inhibitor was given before the cytotoxic drug. We also showed similar schedule-dependent effects when combining AZD1152-HQPA with either oxaliplatin or gemcitabine. A possible explanation lies in the capability of AZD1152-HQPA to induce polyploidy, which should be responsible for the enhanced chemotherapeutics ability to induce apoptosis on the endoreduplicating cells. AZD1152-HQPA exerts its activity through the inhibition of histone 3 phosphorylation even if it also modulates other signal-transduction pathways, such as survival pathways that involve the tumour suppressor protein p53. In the analysis of cell targets modulated by AZD1152, we focused our attention on p53 modulation, and in agreement with Nair *et al* (2009b), we found that AZD1152 was associated with an increase in the expression level of this tumour suppressor protein, but only when it was wild type. Conversely in the pancreatic cancer cell line, with mutated p53, modulation was absent even if its pharmacological treatment, *in vitro* and *in vivo*, was promising.

Our study stresses the importance in defining the best drug sequencing for combined administration to optimise a novel therapeutic approach (Xu *et al*, 2003; Azzariti *et al*, 2006a, 2006b). The possibility to utilise AZD1152-HQPA as enhancer of the effectiveness of oxaliplatin and gemcitabine was verified in colon and pancreatic cancer models, respectively. Promising results, obtained in both models, especially using the schedule in which AZD1152-HQPA was given before gemcitabine or oxaliplatin, support a positive role for AZD1152 as an enhancer of the antitumour effects of standard of care chemotherapy in patients with colorectal and pancreatic cancer. We decided to validate our hypothesis in an animal model starting with the pancreatic cancer model utilising a pancreatic tumour xenograft. Indeed, the consecutive combination of AZD1152 with gemcitabine seems to suggest the possible importance of a sequence-dependent effect because the sequential association was on average more effective than either single-agent therapy in inhibiting and delaying the tumour growth, without inducing important toxicities. Both these promising aspects and the knowledge of specific drug sequencing could be very helpful for the future planning of preclinical and, above all, clinical studies to avoid any failures in the AZD1152 clinical development.

In a recent report, Aihara *et al* (2009) suggested the utilisation of an orthotopic xenograft model instead of or together with a subcutaneous xenograft model because the benefits are easy evaluation of tumour growth and a correlation of the biological response of drugs to the natural tumour microenvironment, respectively. We agree with this idea, and in the future, we will evaluate AZD1152 in combination with gemcitabine in orthotopic xenograft models of pancreatic cancer. However, even if some promising data are available on the preclinical and clinical utilisation of this class of target-oriented drugs, some points to consider include: (1) to further investigate genetic instability in these cancers, which through deregulation of Aurora kinases contributes to the acquisition of genetic aberrations, promoting the development of malignancy; (2) to exactly define the optimal drug schedules to maximise target inhibition; and (3) to identify predictive biomarkers, to select patients for treatment with an Aurora kinase inhibitor, different from phosphorylated histone H3, which is a regulator of mitosis, and thus, it is not a cancer-specific inhibitor in the strictest sense.

As reported, AZD1152 has been already utilised in a phase I clinical trials in patients with advanced solid tumours, in which this drug showed to induce a significant disease stabilisation, and in phase I/II trials in patients with advanced acute myeloid leukaemia showing an acceptable tolerability profile and an overall clinical response rate of 23% (Lowenberg *et al*, 2009; Boss *et al*, 2010). Our opinion is that the *in vitro* and *in vivo* data strongly suggest the use of AZD1152 in the treatment of pancreatic cancer, and these data are consistent with clinical trials already ongoing in pancreatic cancer using drugs inhibiting Aurora kinases, such as *MLN8054* and *AS703569*, selective inhibitors of Aurora A kinase and Aurora B kinase, respectively (www.clinicaltrials.gov).

In agreement with other literature data, our *in vitro* and *in vivo* results point toward additive and synergistic anticancer effects between AZD1152 and conventional chemotherapy, and our hope lies in the possibility to translate such combinations into the clinic.
